# Adaptive discrimination between harmful and harmless antigens in the immune system by predictive coding

**DOI:** 10.1016/j.isci.2022.105754

**Published:** 2022-12-07

**Authors:** Kana Yoshido, Honda Naoki

**Affiliations:** 1 Laboratory of Theoretical Biology, Graduate School of Biostudies, Kyoto University, Yoshida-Konoecho, Sakyo, Kyoto 606-8315, Japan; 2 Laboratory of Data-driven Biology, Graduate School of Integrated Sciences for Life, Hiroshima University, Kagamiyama, Higashi-Hiroshima, Hiroshima 739-8526, Japan; 3 Theoretical Biology Research Group, Exploratory Research Center on Life and Living Systems (ExCELLS), National Institutes of Natural Sciences, Okazaki, Aichi 444-8787, Japan; 4 Kansei-Brain Informatics Group, Center for Brain, Mind and Kansei Sciences Research (BMK Center), Hiroshima University, Kasumi, Minami-ku, Hiroshima 734-8551, Japan

**Keywords:** Immunology, mathematical biosciences, computing methodology, machine learning

## Abstract

The immune system discriminates between harmful and harmless antigens based on past experiences; however, the underlying mechanism is largely unknown. From the viewpoint of machine learning, the learning system predicts the observation and updates the prediction based on prediction error, a process known as “predictive coding.” Here, we modeled the population dynamics of T cells by adopting the concept of predictive coding; conventional and regulatory T cells predict the antigen concentration and excessive immune response, respectively. Their prediction error signals, possibly via cytokines, induce their differentiation to memory T cells. Through numerical simulations, we found that the immune system identifies antigen risks depending on the concentration and input rapidness of the antigen. Further, our model reproduced history-dependent discrimination, as in allergy onset and subsequent therapy. Taken together, this study provided a novel framework to improve our understanding of how the immune system adaptively learns the risks of diverse antigens.

## Highlights

• Mathematical model of immune response by T-cell population dynamics was developed
• Immunological memory is hypothesized to be formed based on predictive coding
• Predictive coding mechanism led to discrimination between harmful/harmless antigens
• The model also reproduced the onset and therapy of allergy

## Graphical abstract

**Figure:**
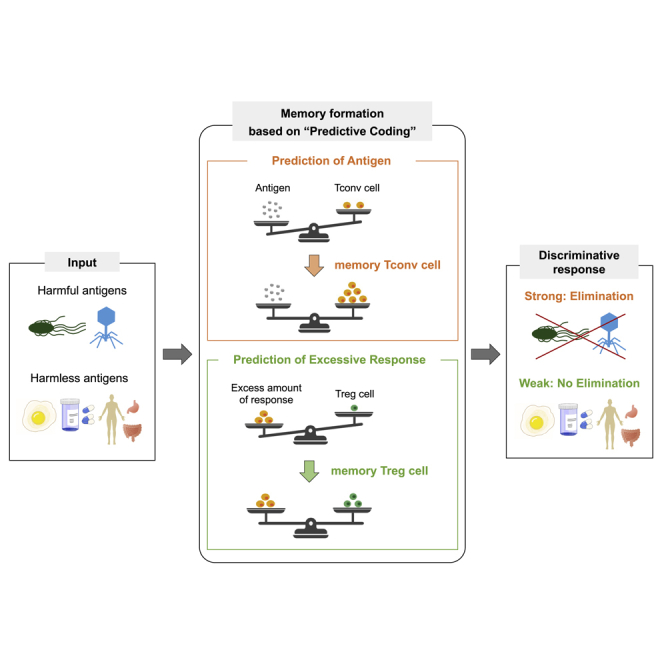

## Introduction

The immune system faces the challenge of identifying unknown risks of diverse antigens and inducing proper immune responses. For harmful antigens, such as pathogens, the immune system induces strong immune responses for their elimination, whereas, for harmless antigens, such as food and self-antigens, it does not lead to strong responses to prevent unnecessary inflammation. Thus, the immune system should discriminate between harmful and harmless antigens appropriately. Defects in this discrimination induce immune diseases, including allergies and autoimmune diseases.^1^^,^^2^ However, the mechanism by which the immune system distinguishes between harmful and harmless antigens upon exposure to numerous antigens remains to be understood. This study aimed to explore this field through computational modeling of T-cell population dynamics and we first introduced into immunology the concept that the immune system predicts its environment using predictive coding.

The central organizers of adaptive immunity are T cells, each of which expresses different T-cell receptors (TCRs) to specifically recognize antigens presented by antigen-presenting cells, such as dendritic cells (DCs).^3^^,^^4^^,^^5^ Through the process of T-cell differentiation, the cells responsive to self-antigens are eliminated^6^^,^^7^^,^^8^; however, there still remain those that are specific not only to harmful antigens but also to harmless ones. Namely, such T cells have no way of knowing whether the antigen is harmful or harmless. Nevertheless, the immune system responds strongly to harmful foreign antigens but not to harmless ones. Therefore, we focused on the fact that the antigen specificity of T cells cannot explain the mechanism by which the immune system discriminates between harmful and harmless antigens.

**Figure 1:**
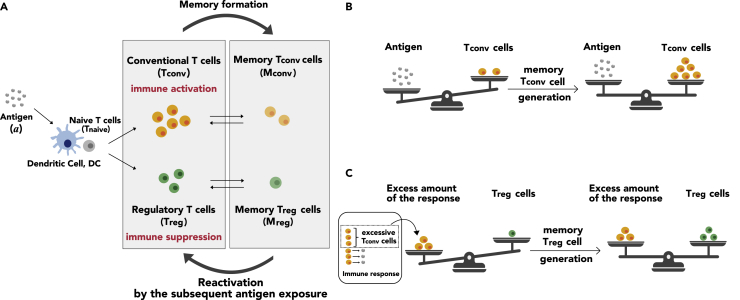
Scheme of the predictive immune memory model (A) Population dynamics model of T cells in response to antigen input. The model includes the differentiation of T_naive_ cells into T_conv_ and T_reg_ cells by antigen-presenting cells such as dendritic cells (DCs), the differentiation of T_conv_ and T_reg_ cells into memory T cells, and the reactivation of memory T cells into T cells upon subsequent exposure to antigens. (B and C) Predictive coding-based immunological memory formation. (B) Generation of memory T_conv_ cells. Memory T_conv_ cells are generated based on the prediction error $e_{c}{\left|a-m_{c}T_{conv}\right|}_{+}$. In other words, the production of memory T_conv_ cells is induced when the concentration of antigens is excessive compared to that of T_conv_ cells in order to efficiently eliminate antigens. (C) Generation of memory T_reg_ cells. Memory T_reg_ cells are generated based on the prediction error $e_{r}{\left|g\left(T_{conv}\right)-a-m_{r}T_{reg}\right|}_{+}$. In other words, the production of memory T_reg_ cells is induced when the excess amount of response, evaluated by the difference between the intensity of T_conv_ cell activation ($g\left(T_{conv}\right)$) and antigen concentration, is larger than the concentration of T_reg_ cells in order to prevent unnecessary inflammation.

The immune response is organized by the population dynamics of various cell types (Figure 1A). It is initiated by antigen-presenting cells, such as DCs, which take in antigens and present them to T cells. Naive T (T_naive_) cells, with TCRs on their surface, recognize specific antigens presented by DCs. T_naive_ cells then differentiate into various types of T cells, such as conventional T (T_conv_) cells and regulatory T (T_reg_) cells, depending on cytokines, such as interleukins (ILs), in their microenvironment.^9^ T_conv_ and T_reg_ cells play distinct roles in immune responses; T_conv_ cells, including T-helper (Th) 1, Th2, Th17 cells, accelerate immune responses, leading to the elimination of antigens by activating downstream cells, such as B cells and killer T cells,^9^^,^^10^^,^^11^ whereas T_reg_ cells work as a brake for immune responses via the regulation of DCs and suppressive cytokines.^12^^,^^13^ Note that this kind of T_reg_ cells is called induced T_reg_ cells, and their differentiation pathway is different from that of naturally occurring T_reg_ cells, which specifically respond to self-antigens. Following the immune response, most of the induced T_conv_ and T_reg_ cells are removed by apoptosis^14^^,^^15^; however, a small population of them differentiates into memory T cells, namely memory T_conv_ cells and memory T_reg_ cells, and they persist in the body for a long time.^16^^,^^17^ Upon subsequent encounters with the same antigen, memory T cells are rapidly activated, resulting in more efficient responses.^18^^,^^19^ This is termed immunological memory. Thus, the combinatorial dynamics of various types of T cells determine the intensity of the immune response. In other words, the discrimination between harmful and harmless antigens must be achieved at the level of T-cell population dynamics.

Discrimination between harmful and harmless antigens for each antigen is not always constant and varies in antigen experience-dependent manner. A prominent example is the onset and therapy of allergy, which is defined as an excessive response to harmless antigens, including pollen and mites. Although allergens, defined as substances that cause allergy, are initially regarded as harmless in our body, response to them can intensify upon repeated exposures, leading to allergic symptoms. Such a change in responsiveness indicates that the immune discrimination of allergens can change from harmless to harmful. Furthermore, allergic symptoms, the immune responses to allergens, can be weakened by allergen immunotherapy,^20^^,^^21^^,^^22^ in which a small amount of allergen extract (not enough to cause symptoms) is repeatedly administered to the patients; after the therapy, allergic symptoms do not occur even when patients are exposed to large amounts of the allergen. This means that discrimination can be reversed from harmful to harmless through allergen immunotherapy. Thus, the immune system adaptively changes discrimination depending on the temporal history of antigens. Experimentally and clinically, allergen immunotherapy has been reported to induce regulatory cell populations, such as T_reg_ cells, and suppressive cytokines, such as IL-10.^23^^,^^24^^,^^25^ However, the mechanisms by which immune discrimination is adaptively updated by antigen experience largely remain unclear.

The immune system can be viewed as an adaptive learning system that updates the discrimination of antigen risk. To induce the most appropriate responses, the immune system needs to predict and prepare for the subsequent invasion of antigens by the formation of memory cells. From the perspective of the machine learning theory, a more accurate prediction is achieved by repeated observation and prediction, in which the prediction is updated based on prediction error, which is the difference between observation and prediction. This concept, called “predictive coding,” was originally proposed in neuroscience^26^ and has been widely accepted as a guiding principle for understanding learning systems, such as brain and artificial intelligence.^27^^,^^28^^,^^29^ In this study, we adopted this concept to understand the immune system as a learning system. We hypothesized that T_conv_ and T_reg_ cells predict the risk of antigens and excessive response, respectively, and their predictions can be updated by prediction errors via the production of memory T cells.

Based on the idea of predictive coding, this study aimed to address how the immune system discriminates between harmful and harmless antigens and how it changes its response depending on the history of antigens. We developed a mathematical model of antigen-induced T-cell population dynamics named “the predictive immune memory model.” By simulating the model, we demonstrated that the immune system can discriminate between harmful and harmless antigens using the predictive coding mechanism in an antigen concentration- and input rapidness-dependent manner. The model also demonstrated antigen history-dependent immune discrimination, as seen in the onset and therapy of allergy. Furthermore, we found that the dose-response of T-cell activation does not affect the outcome of allergen immunotherapy but changes its persistence upon additional higher exposure to allergens.

## Results

### Mathematical model for T-cell population dynamics

To examine how the immune system discriminates between harmful and harmless antigens at the level of the T-cell population, we developed a mathematical model for the population dynamics of T cells and named it “the predictive immune memory model” (Figure 1A). The model consists of T_conv_, T_reg_, and their memory cells. T_conv_ and T_reg_ cells are generated by the differentiation of T_naive_ cells, activation of memory T cells, and their proliferation, as shown later in discussion.

(Equation 1) $$\begin{array}{l} \frac{d}{dt}T_{conv}=-d_{c}T_{conv}+\frac{D_{c}}{1+s_{r}T_{reg}}T_{conv}+k_{c}T_{naive}a+w_{c}M_{conv}a-E_{c}T_{conv}\text{,} \end{array}$$

(Equation 2) $$\begin{array}{l} \frac{d}{dt}T_{reg}=-d_{r}T_{reg}+\frac{D_{r}}{1+s_{c}T_{conv}}T_{reg}+k_{r}T_{naive}a+w_{r}M_{reg}a-E_{r}T_{reg}\text{,} \end{array}$$

where $T_{conv}$ and $T_{reg}$ represent the populations of T_conv_ and T_reg_ cells, respectively; $M_{conv}$ and $M_{reg}$ represent the populations of memory T_conv_ and memory T_reg_ cells, respectively; $T_{naive}$ indicates a positive constant which represents the population of T_naive_ cells; a represents the concentration of antigen input; $d_{i}$, $k_{i}$, and $w_{i}$ (i∈{c,r}) indicate the rates of death due to apoptosis, differentiation from T_naive_ cells, and production of T cells from memory T cells, respectively. In addition, the second terms represent the proliferation of T_conv_ and T_reg_ cells, which are inhibited by each other through some possible mechanisms, such as the competition for limited sources of cytokines (IL-2) and contact with DCs,^30^^,^^31^^,^^32^ where $D_{i}$ and $s_{i}$ (i∈{c,r}) represents proliferation rate of each T cell itself and the rate of suppression to the counterparts, respectively. The fifth terms represent the decrease of T cells by their differentiation into memory T cells, as described later in discussion. Memory T cells differentiate from T_conv_ and T_reg_ cells as

(Equation 3) $$\begin{array}{l} \frac{d}{dt}M_{conv}=-d_{mc}M_{conv}+E_{c}T_{conv}\text{,} \end{array}$$

(Equation 4) $$\begin{array}{l} \frac{d}{dt}M_{reg}=-d_{mr}M_{reg}+E_{r}T_{reg}\text{,} \end{array}$$

where $d_{mc}$ and $d_{mr}$ indicate the death rates of memory T_conv_ and memory T_reg_ cells, respectively. We regarded their death rates as zero in the time span of our simulations due to the longevity of memory T cells ($d_{mc}=d_{mr}=0$). Note that $E_{c}$ and $E_{r}$ are not constant parameters but are situation-dependent, following the idea of predictive coding (see the next section for details). In this model, we defined the intensity of response R, which is positively and negatively regulated by T_conv_ and T_reg_ cells, respectively, as

(Equation 5) $$\begin{array}{l} \frac{d}{dt}R=-\left(r_{0}+r_{s}T_{reg}\right)R+r_{a}T_{conv}, \end{array}$$

where $r_{0}$ indicates a positive constant, which causes the convergence of R to zero in the absence of T_conv_ and T_reg_ cells; $r_{a}$, and $r_{s}$ indicate the activation rates by T_conv_ cells and suppression rates by T_reg_ cells, respectively. Although we artificially defined the intensity R, we can biologically interpret $r_{0}$ as the amounts of other types of T_reg_ cells called naturally occurring T_reg_ cells, which possibly contribute to the suppression of excessive inflammation, and $r_{a}$ and $r_{s}$ could correspond to the amount of cytokines from T_conv_ cells which activate the response and those from T_reg_ cells which suppress the response, respectively.

### Predictive coding scheme

We have introduced the concept of predictive coding under the hypothesis that the immune system predicts the level of antigen exposure and its consequent inflammation in an antigen experience-dependent manner. More specifically, the predictive coding scheme states that T_conv_ and T_reg_ cells are predictors of the antigen amount and excess amount of immune response, respectively, and that their predictions are updated based on prediction errors via the formation of memory T_conv_ and memory T_reg_ cells.

Since T_conv_ cells are the control center to achieve antigen elimination by inducing downstream reactions, they must be adequately controlled depending on the change in antigen concentration; when the concentration of antigens is excessive compared to that of T_conv_ cells, more T_conv_ cells need to be generated to completely eliminate the antigens in our hypothesis (Figure 1B). Accordingly, the production rate of memory T_conv_ cells can be described by

(Equation 6) $$\begin{array}{l} E_{c}=e_{c}{\left|a-m_{c}T_{conv}\right|}_{+}\text{,} \end{array}$$

where $e_{c}$ and $m_{c}$ indicate positive constants and ${\left|x\right|}_{+}$ represents ramp function (i.e., ${\left|x\right|}_{+}=0\;\left(x<0\right),x\;\left(x≧0\right)$). Note that $E_{c}$ is the prediction error of antigen concentration, since a and $m_{c}T_{conv}$ represent the observation and prediction of the antigen concentration, respectively. Thus, memory T_conv_ cells are upregulated by the prediction error $E_{c}$ (Equation 3).

On the other hand, T_reg_ cells play an important role in the prevention of excessive immune responses. Thus, their amount should be regulated based on the intensity of the response; when the excess amount of the immune response is larger than the concentration of T_reg_ cells, more T_reg_ cells need to be generated to suppress the excessive immune responses in our hypothesis (Figure 1C). Therefore, the production rate of memory T_reg_ cells can be described by

(Equation 7) $$\begin{array}{l} E_{r}=e_{r}{\left|f\left(T_{conv},a\right)-m_{r}T_{reg}\right|}_{+}\text{,} \end{array}$$

where $e_{r}$ and $m_{r}$ indicate positive constants, and $f\left(T_{conv},a\right)=g\left(T_{conv}\right)-a$ represents the excess amount of the immune response compared to antigen concentration. Here, we assumed that T_reg_ cells evaluated the level of T_conv_ cell activation by $g\left(T_{conv}\right)=A_{\max}T_{conv}/\left(T_{conv}+K\right)$, where $A_{\max}$ and K indicate positive constants. Note that $E_{r}$ is the prediction error of the excess amount of immune response, since $f\left(T_{conv},a\right)$ and $m_{r}T_{reg}$ represent the observation and prediction of the excess amount of immune response, respectively. Thus, memory T_reg_ cells were upregulated by the prediction error $E_{r}$ (Equation 4). Notably, we hypothesized that the generation of memory T cells (not T cells) reflects the calculation of the prediction error since memory T cells rather than T cells remain for a long time serving as immunological memory.

As an implementation of memory formation based on predictive coding, we assumed that the calculation of predictive coding can be achieved by cytokines. Cytokines secreted from immune cells determine their differentiation and proliferation under communication across various types of immune cells.^9^^,^^33^ In this study, we regarded cytokines as the medium for transmitting this quantitative information. Specifically, the amounts of T_conv_ and T_reg_ cells can be coded by the concentration of cytokines secreted by themselves, whereas the amounts of antigens can be coded by cytokines secreted from antigen-presenting cells, such as DCs and macrophages. Based on these information-carrying cytokines, we hypothesized that the information obtained from each kind of cytokines is integrated into T cells and that prediction errors are computed through intracellular signal transduction in T cells. The parameters used in the numerical simulations are provided in Table S1. Although all of the parameters were just our assumption, we validated them by the parameter sensitivity analysis (Figures S1 and S2).

### Concentration-dependent discrimination between harmful and harmless antigens

**Figure 2:**
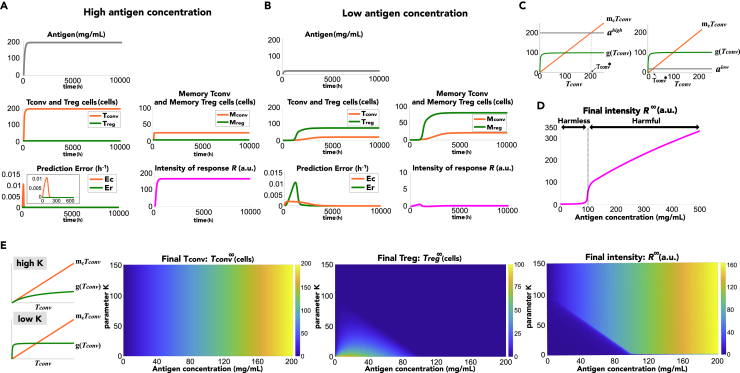
Antigen concentration-dependent immune discrimination (A and B) Immune responses simulated with the exposure of (A) high and (B) low concentration of antigens. The inset shows an enlarged view of the prediction error in the early phase. (C) Diagram for predictive coding-based memory formation. Antigen concentration is predicted by T_conv_ cells (orange line). When antigen concentration is observed (gray line), positive prediction error ($a-m_{c}T_{conv}$) increases memory T_conv_ cells, until $T_{conv}$ converges to ${T_{conv}}^{*}$ at the intersection of orange and gray lines. On the other hand, T_reg_ cells are assumed to predict the excess amount of immune response. The green line indicates the intensity of T_conv_ cell activation evaluated by T_reg_ cells. The excess amount of immune response is evaluated by the difference between this intensity of T_conv_ cell activation and observed antigen concentration. When antigen concentration is observed (gray line), positive prediction error between observed excess amount of immune response ($g\left(T_{conv}\right)-a$) and its prediction ($m_{r}T_{reg}$) increases memory T_reg_ cells, until the prediction error converges to zero. The left and right panels show the diagram for the case in which the observed antigen concentrations are high and low, respectively. Notably, different formulations of T_conv_ cell activation evaluated in memory T_conv_ and T_reg_ cell formation (orange and green lines, respectively) is necessary to the accumulation of memory T_reg_ cells under the exposure of low concentration of antigens. (D) Change in intensity of immune responses depending on antigen concentrations. Convergence value of the intensity R is plotted upon the steady exposure to each antigen concentration. (E) Steady-state responses of T_conv_ and T_reg_ populations and the immune intensity *R* depending on antigen concentration and a parameter *K* in $g\left(T_{conv}\right)$. Left panels visually represent how the parameter K in $g\left(T_{conv}\right)$ changes the diagram for predictive coding-based memory formation as in (C), where we can regard $m_{c}T_{conv}$ (orange line) and $g\left(T_{conv}\right)$ (green line) as the evaluation of T_conv_ cell activation in memory T_conv_ and memory T_reg_ cell generation, respectively.

To examine the difference between harmful and harmless antigens for the immune system, we focused on the effect of antigen concentration on the immune response. We simulated the model with high and low concentrations of antigen input (Figures 2A and 2B) and found that the steady exposure of high and low concentrations of antigens caused more accumulation of memory T_conv_ and memory T_reg_ cells, respectively. At high antigen concentrations (Figure 2A), memory T_conv_ cells were generated until the prediction error $e_{c}{\left|a-m_{c}T_{conv}\right|}_{+}$ was minimized to zero. Memory T_reg_ cells were not generated, since the prediction error $e_{r}{\left|g\left(T_{conv}\right)-a-m_{r}T_{reg}\right|}_{+}$ was always zero (left panel in Figure 2C). Therefore, the intensity of immune response R converged to a high level. On the other hand, at low antigen concentrations (Figure 2B), memory T_conv_ cells were produced, similar to the exposure of high antigen concentration. Memory T_reg_ cells were generated more since the prediction error $e_{r}{\left|g\left(T_{conv}\right)-a-m_{r}T_{reg}\right|}_{+}$ was positive, and the generation of memory T_reg_ cells continued until the prediction error was minimized to zero (right panel in Figure 2C). Therefore, the intensity of immune response was low. To summarize the immune responses depending on antigen concentrations, there was threshold of the antigen concentration (a≒100), indicating that immune responses were specifically suppressed under low concentration of antigen exposures (Figure 2D). These results suggested that the immune system with predictive coding discriminates between harmful and harmless antigens based on antigen concentration.

Next, we examined the conditions necessary for the immune system to properly distinguish between harmful and harmless antigens depending on their concentration. We performed antigen concentration-dependent simulations by varying the parameter K, which regulated the T_reg_ cell-estimated level of T_conv_ cell activation in memory T_reg_ generation (left panels in Figure 2E). We found that antigen discrimination could be achieved only with low K, in which immune responses were specifically suppressed under low concentration of antigen exposures (Figure 2E). Because T_reg_ cells underestimated and overestimated the level of T_conv_ cell activation in low antigen concentration with high and low K, respectively (left panels in Figure 2E), this result suggested that suppressive immune responses at low concentration of antigens can be achieved by the overestimation of T_conv_ cell activation in memory T_reg_ generation compared to the estimation in memory T_conv_ generation at low antigen concentration. Note that the relative values of K and $m_{c}$ strictly determine whether antigen concentration-dependent discrimination is achieved since the overestimation of T_conv_ cell activation is defined by the relative estimation of T_conv_ cell activation in memory T_conv_ cell generation and that in memory T_reg_ cell generation. This also means that once the overestimation of T_conv_ cell activation in memory T_reg_ cell generation is satisfied by K and $m_{c}$, antigen concentration-dependent discrimination (seen in Figure 2D) can be robustly achieved without depending on other parameters, which we also verified by the parameter sensitivity analysis (Figures S1 and S2).

### Input rapidness-dependent discrimination between harmful and harmless antigens

**Figure 3:**
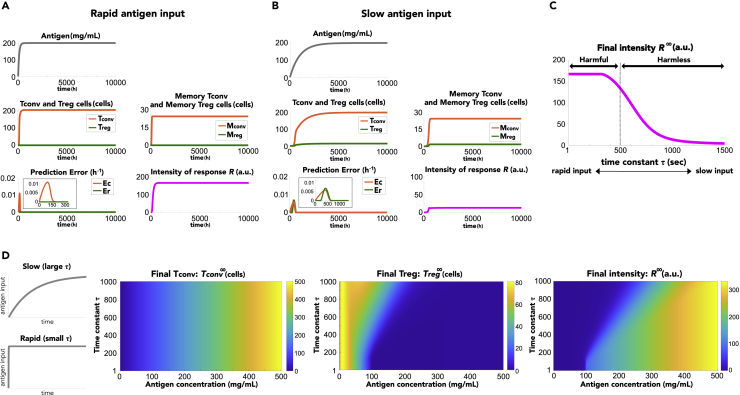
Antigen input rapidness-dependent immune discrimination (A and B) Immune responses to (A) rapid and (B) slow inputs of high antigen concentration. Antigens were administered as $a\left(t\right)=a_{0}\left(1-e^{-t/\tau }\right)$, where τ indicates the time constant. Insets show an enlarged view of the prediction error in the early phase. (C) Change in intensity of immune responses depending on time constants of antigen administration. Convergence value of the intensity *R* is plotted at each time constant. High antigen concentrations ($a_{0}=200$) were administered. (D) Steady-state response of T_conv_ and T_reg_ populations and the immune intensity *R* depending on antigen concentration $a_{0}$ and time constant τ. Left panels visually represent how time constant τ affects rapidness of antigen inputs.

We focused on the rapidness of antigen input (the speed of antigen input) as another possible factor for discrimination between harmful and harmless antigens. We simulated the model in response to antigen inputs with different time constants (Figures 3A and 3B). Similar to that in Figure 2A, the intensity of immune response was high upon rapid exposure to high concentrations of antigens (Figure 3A). However, when the concentration of antigens increased slowly, eventually reaching a high concentration, the intensity of the immune response became weaker (Figure 3B). This was because the slowly increasing antigen input enabled the immune system to have a longer experience of low antigen concentration before reaching a high concentration, which caused a positive prediction error in memory T_reg_ cell generation followed by the production of memory T_reg_ cells.

Next, we examined input rapidness-dependent immune responses under exposure to the same high concentration of antigens with different input time constants and found that there was a threshold of time constant for discrimination between harmful and harmless antigens (τ≒500) (Figure 3C). This result showed that even when the final concentration was high, the immune system could recognize the antigens with slow input as harmless. To summarize the results, we examined the immune responses depending on both the antigen concentration and its input rapidness (Figure 3D). We found that low concentrations of antigens induced suppressive responses independent of their input rapidness. On the other hand, high concentrations of antigens induced responses with different intensities depending on their input rapidness; the immune system caused strong responses to rapidly increasing antigens while it caused suppressive responses to slowly increasing antigens. In addition, we examined immune discrimination with a time delay in memory formation (see the STAR Methods section) and demonstrated that the discrimination between harmful and harmless antigens based on the antigen concentration and its input rapidness was similarly achieved with a time delay in memory formation (Figures S3 and S4). Together, these results suggested that the immune system discriminates between harmful and harmless antigens based on their input rapidness as well as their concentration.

### History-dependent discrimination between harmful and harmless antigens

**Figure 4:**
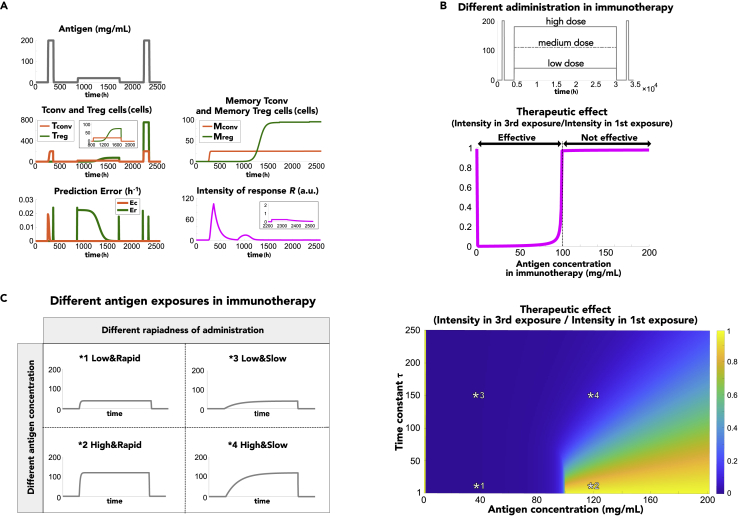
Antigen history-dependent immune discrimination in allergen immunotherapy (A) Temporal change in immune responses to a series of different antigen inputs. The first antigen input was high enough for the induction of allergy, the second one was applied for allergen immunotherapy, and the third one was for checking the therapeutic effect. Insets show an enlarged view of the populations of T_conv_ and T_reg_ cells during allergen immunotherapy and the intensity of the response during the third antigen input. (B) Therapeutic effects depending on antigen concentration administered during allergen immunotherapy. Therapeutic effect was evaluated by the ratio of maximum immune intensity *R* in the third antigen input to that in the first antigen input. A ratio smaller than one indicates the success of the therapy. Upper panel shows the schedule of antigen inputs with different doses in allergen immunotherapy. (C) Effect of allergen immunotherapy depending on antigen concentration and rapidness of antigen inputs during allergen immunotherapy. The left diagram shows examples of antigen input in allergen immunotherapy with different concentrations and rapidness, corresponding to asterisks in the right panel.

Discrimination between harmful and harmless antigens is not invariable throughout our life span, in other words, discrimination can change depending on experiences of antigen exposure: antigen history. For example, at the onset of allergy, discrimination of the same antigen changes from harmless to harmful, whereas its discrimination can be reversed by allergen immunotherapy. To examine the mechanism of antigen history-dependent changes in immune discrimination, we simulated the immune responses to successive but different patterns of antigen exposure (Figure 4A). Specifically, we applied rapid exposure to high concentrations of antigens inducing allergy, followed by exposure to low concentrations of antigens, as allergen immunotherapy, and subsequently, rapid exposure to high concentrations of antigens again. The final input was provided to examine the effect of allergen immunotherapy.

After the first exposure to high concentrations of antigens, a strong immune response was induced due to the positive prediction error in memory T_conv_ cell generation, as shown in antigen concentration- and input rapidness-dependent discrimination. Upon exposure to a low concentration of antigens thereafter, more T_conv_ cells were produced than T_reg_ cells at the initiation of therapy due to the accumulated memory T_conv_ cells. In contrast, T_reg_ cells were gradually generated since a low concentration of antigens achieved a positive prediction error in memory T_reg_ cell generation. Accordingly, exposure to low concentrations of antigens for a certain period of time enabled the accumulation of memory T_reg_ cells. Therefore, even when the immune system was exposed to high concentrations of antigens again, more T_reg_ cells were generated, and the intensity of the immune response became weak. This result indicated that the simulation successfully reproduced the immune response at the onset of allergy and the effect of allergen immunotherapy. In summary, the immune system could discriminate between harmful and harmless antigens upon the first exposure to antigens in an antigen concentration- and input rapidness-dependent manner. Furthermore, the discrimination could adaptively change due to memory formation, based on predictive coding, in an antigen history-dependent manner.

Furthermore, we examined how therapeutic strategies influence the effect of allergen immunotherapy by evaluating the ratio of maximum intensity R in response to antigen input after therapy to that before therapy. We found that allergen immunotherapy was effective only when a low antigen dose was administered for the therapy (Figure 4B), which is consistent with the accumulation of memory T_reg_ cells at low antigen concentrations, as shown in Figure 2. Additionally, we examined the effect of both antigen concentration and input rapidness in allergen immunotherapy on the therapeutic effect (Figure 4C) and found that a low concentration and/or slow input enabled effective allergen immunotherapy. These findings could explain the validity of therapeutic strategies currently used in numerous clinical settings where antigen administration is initiated at a low dose and then gradually increased in the early phases of allergen immunotherapy with the aim of avoiding allergic symptoms during the therapy.^34^^,^^35^^,^^36^

### The property of T-cell activation affects history-dependent discrimination

**Figure 5:**
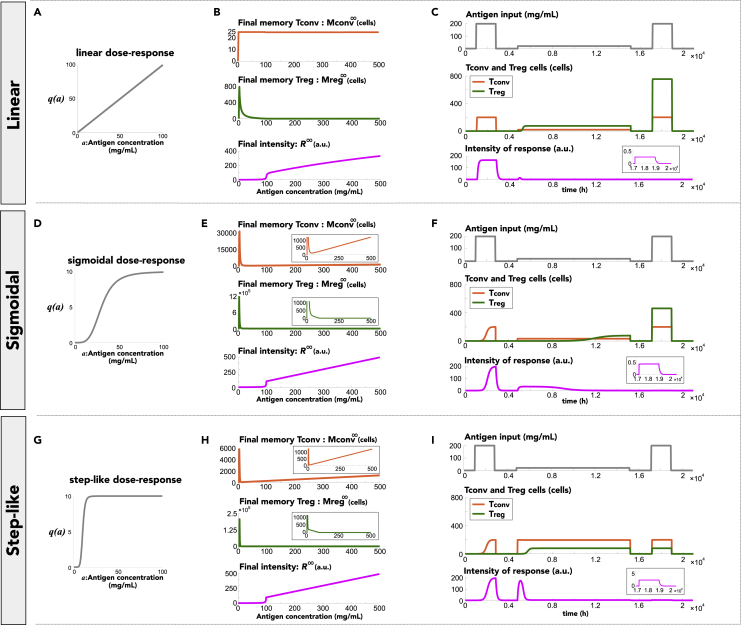
Robustness of allergen immunotherapy effect against the property of T-cell activation (A, D, and G) Three types of dose-responses of T-cell activation to antigen concentrations. q(a) represents the dose-response curves of T_conv_ and T_reg_ cell activation. (B, E, and H) Change in memory T_conv_ and memory T_reg_ cell accumulation and the intensity of immune responses, depending on antigen concentrations. The convergence values of memory T_conv_ and memory T_reg_ cell populations and the intensity are plotted upon steady exposure to each antigen concentration. Insets in (E) and (H) show an enlarged view of memory T_conv_ and memory T_reg_ cell accumulation. (C, F, and I) Temporal change in immune responses before, during, and after the allergen immunotherapy (as in Figure 4). Insets show an enlarged view of the intensity of the responses during the third antigen input.

So far, the model has assumed that T-cell activation is linearly associated with antigen concentration. However, antigen concentration-dependent of T-cell activation might follow various types of activation patterns since the difference in ligands and its consequent difference in binding properties to TCRs largely affect the T-cell activation potency.^37^^,^^38^^,^^39^ Thus, we examined the effect of the dose-response pattern of T-cell activation on immune discrimination (see the STAR Methods section). Here, we simulated the model with three types of dose-response curves (linear, sigmoidal, and step-like curves) for both T_conv_ and T_reg_ cells (Figures 5A, 5D, and 5G). We found that different dose-response types of T-cell activation induced different accumulation patterns of memory T_conv_ cells depending on antigen concentrations (top panels in Figures 5B, 5E, and 5H). In the case of the linear dose-response curve, memory T_conv_ cells accumulated to an approximately constant value, independent of antigen concentration, while it transiently peaked and then constantly increased with the antigen concentration in cases of the sigmoidal and step-like curves. In contrast, the three dose-response types of T-cell activation did not show a critical difference in the accumulation of memory T_reg_ cells (middle panels in Figures 5B, 5E, and 5H), and antigen concentration-dependent discrimination was achieved in all dose-response types (bottom panels in Figures 5B, 5E, and 5H). Thus, these results indicated that the types of dose-response, or the properties of T-cell activation largely affected memory T_conv_ cell accumulation.

Next, we examined the effect of dose-response types of T-cell activation on history-dependent discrimination by simulating allergen immunotherapy, as shown in Figure 4 (Figures 5C, 5F, and 5I). We found that allergen immunotherapy was successfully achieved in all three dose-responses, since the administration of low concentrations of antigens caused a positive prediction error in memory T_reg_ cell generation (details in Figures S5–S7). However, in the step-like dose-response, the intensity of the response was high at the initiation of allergen immunotherapy due to the production of T_conv_ cells from memory T_conv_ cells, which did not depend on the antigen concentration above the threshold. This implied that some patients with allergy with a step-like dose-response might exhibit allergic symptoms at the early stage of therapy.

From a clinical viewpoint, it is important to discern whether the effect of therapy is persistently maintained against subsequent exposures to antigens. To examine the persistence of the therapeutic effect, we considered the case where, after therapy, patients were exposed to an additional higher concentration of antigens followed by a subsequent lower concentration of antigens (top panels in Figures S5–S7). The final antigen input was applied to quantify the persistence of the therapeutic effect. We found that allergen immunotherapy was effective in all combinations of dose-response types for T_conv_ and T_reg_ cells (Figures S5–S8A). However, its effect was persistently maintained against additional exposures to higher concentrations of antigens only when the dose-response type of T_conv_ cells was linear (Figures S5–S8B). These results indicated that the long-term effect of allergen immunotherapy can be determined by the dose-response type of T_conv_ cell activation. This may explain the heterogeneous effects of allergen immunotherapy across patients, as seen in some cases where patients demonstrated allergic symptoms again after discontinuing allergen immunotherapy.^34^^,^^35^

## Discussion

In order to understand how the immune system discriminates between harmful and harmless antigens despite their diversity, we developed a generalized model that does not assume any prior information on whether each antigen is harmful or harmless. We assumed predictive coding in T-cell population dynamics, by which we first introduced into immunology the concept that the immune system predicts its environment. Specifically, we developed a mathematical model of T-cell population dynamics under the hypothesis that T_conv_ and T_reg_ cells are predictors of the risk of antigens and excessive immune response, respectively, and their responses are regulated by prediction errors via memory T-cell generation. This predictive immune memory model led to both antigen concentration- and input rapidness-dependent discrimination between harmful and harmless antigens. In addition, our model showed that such discrimination can change in an antigen history-dependent manner, as seen in the onset of allergy and its subsequent therapy. To the best of our knowledge, this is the first learning system-based model of discrimination between harmful and harmless antigens by the immune system facing diverse antigens. Furthermore, it could be possible to validate our model in the future through the quantification of T cells with each TCR using single-cell RNA sequencing techniques.

Phenomenologically, harmful antigens usually originate from bacteria and viruses and show a rapid exponential increase in their population once they invade the body. In contrast, harmless antigens, such as food, do not sharply increase in amount inside the body but are expected to change gradually over time. Such distinct characteristics of harmful and harmless antigens can be distinguished by antigen concentration- and input rapidness-dependent discrimination (Figures 2 and 3). Clinically, immune discrimination for the same antigen is known to change over time; for example, the onset of allergy due to exposure to high concentrations of antigens and its remission through allergen immunotherapy. This can be represented by antigen history-dependent discrimination (Figure 4).

In this study, we introduced various types of T-cell activation dose-responses into the model based on the fact that the difference in ligands and its consequent difference in binding properties to TCRs largely affect T-cell activation potency.^37^^,^^38^^,^^39^ Our results showed that the dose-response types of T-cell activation influenced antigen history-dependent changes in the immune response, as seen in allergen immunotherapy and subsequent recurrence (Figures 5, S5, S6, S7, and S8). Overall, these results suggested that the various dose-responses of T-cell activation cause heterogeneity in the immune responses of individuals and/or types of antigens. In fact, some patients with allergy acquire persistent remission of the symptoms by allergen immunotherapy, while others exhibit the symptoms again despite therapy.^34^^,^^35^ Moreover, allergens, such as food and bee venom, sometimes induce lethal symptoms, while others, such as pollens, rarely do so.^40^

Our model is a minimal model that describes essential immune processes at the level of T cells, including antigen presentation by DCs, differentiation from T_naive_ cells to T cells, reactivation of memory T cells to T cells, and memory formation. Although there are several subtypes of T_conv_ cells, such as Th1, Th2, and Th17, which induce different downstream responses, we integrated these subtypes into a single T_conv_ cell population, since all T_conv_ cell subtypes have almost the same role in terms of the elimination of target antigens via different mechanisms.

Downstream of T_conv_ and T_reg_ cells, various types of cells are involved, such as killer T cells, B cells, macrophages, neutrophils, eosinophils, basophils, natural killer T cells, and mast cells. Although we need to consider these various immune cells to discuss the whole immune response, in principle, each subtype of T_conv_ cells facilitates the activation of these downstream cells, while T_reg_ cells suppress the response,^9^^,^^11^ and global activity of these downstream T cells and cytokines should determine the intensity of response. Therefore, our model simply assumed that the intensity of immune responses can be evaluated only by the amounts of T_conv_ and T_reg_ cells.

Some immune cell populations that eliminate antigens, such as killer T cells, T_conv_ cells, B cells, and natural killer T cells, are known to persist in the body for a long time, preparing for a second infection following the first antigen experience by natural infection and vaccination.^41^ Furthermore, previous studies have extensively studied whether regulatory immune cell subsets, such as T_reg_ cells, generate memory populations after antigen exposure.^42^ Due to the lack of memory-specific phenotypic markers for the identification of these populations, it remains controversial whether distinct memory subsets contribute to the persistence of immunosuppressive effects.^43^^,^^44^ However, several studies have defined memory T_reg_ cells and revealed their characteristics as memory populations.^15^^,^^45^ Hence, our model included the memory T_reg_ population as one of the possible implementations of regulatory memory formation.

In this study, we assumed memory T-cell production based on predictive coding. For implementation, we regarded cytokines as the media for transmitting quantitative information. Specifically, the amounts of T_conv_ and T_reg_ cells could be coded by the concentration of cytokines secreted by themselves, whereas the amount of antigens could be coded by the concentration of cytokines secreted from antigen-presenting cells, such as DCs and macrophages. Based on such information-carrying cytokines, we hypothesized that prediction errors in predictive coding can be computed through intracellular signal transduction in T cells. Similar to our hypothesis, this type of quantitative function of cytokines has recently become a point of focus, although qualitative molecular discoveries have been traditionally explored, such as the identification of previously unknown cytokines and potential T_conv_ cell subsets. For instance, various experimental and computational studies revealed that immune cell activation was controlled by cell density via cytokines.^32^^,^^46^^,^^47^^,^^48^^,^^49^^,^^50^ This phenomenon is called quorum sensing and was originally proposed in bacterial cells^51^^,^^52^ and then adopted to elucidate immune dynamics.^53^^,^^54^^,^^55^ Our hypothesis that memory formation based on the calculation of T-cell populations can be achieved by cytokines is consistent with the concept of quorum sensing in terms of cell density-dependent induction of responses achieved by cytokines. Notably, it was also suggested that T_conv_ cell density regulated the rate of memory differentiation.^56^ Furthermore, we introduced the idea that the information on cell densities was integrated into T cells because it is possible that T cells sensitive to various cytokines can integrate signals from them. To validate this hypothesis, however, it is necessary to quantify the time series of T-cell populations with each TCR and cytokines in future experiments.

Appropriate immune responses to each antigen kind have traditionally been assumed to be achieved at the single-cell level due to the antigen specificity of TCRs on each T cell. In addition, it has been suggested that the antigens themselves can determine the responses, which is referred to as the “danger theory.”^57^^,^^58^^,^^59^ The theory states that T cells are activated only in the presence of danger signals, such as pathogen-associated molecular patterns (PAMPs),^60^ because they upregulate the expression of costimulatory molecules on antigen-presenting cells. However, this kind of antigen-type-dependent immune response does not explain the temporal change of immune discrimination (i.e., immune activation by and tolerance to the same antigen). It also does not explain the immune responses to harmless antigens, as seen in allergy and autoimmune diseases. In addition, some studies examining the quorum sensing mechanism suggested that the state of the cell population level, such as their densities and distributions, has a more important role in regulating immune responses than distinct antigen properties.^53^^,^^54^^,^^55^ Therefore, our model hypothesized that memory T cells were generated based on the calculation of antigen concentrations and T-cell populations. This hypothesis is also based on the concept of immune regulation, which does not premise prior information on the risk of antigens and their own properties.

Several studies have reported computational models of immune dynamics. Different models of T-cell population dynamics have focused on allergen immunotherapy. In one model, allergen immunotherapy was represented by prolonged activation of T_reg_ cells with a large time constant.^61^ In another model, the effect of allergen immunotherapy was represented by a transition from a Th2 cell-dominant state to a T_reg_ cell-dominant state.^62^ However, the effect of the therapy spontaneously disappeared after antigen elimination due to the absence of explicit T-cell memory.

Immune discrimination had earlier been assessed by various mathematical models. Sontag modeled the interaction between T-cell population and antigens, such as pathogens and tumor cells^63^; this study revealed immune discrimination based on dynamic features of antigen presentation, such as the growth rate of antigens. In addition, Pradeu et al. proposed the discontinuity theory stating that discontinuous (sudden or intermittent) exposures to antigens induce vigorous immune responses, whereas progressive and persistent exposures induce weak responses.^64^ The findings of these studies were consistent with our results in terms of immune discrimination being independent of antigen type; however, they lacked immunological memory formation.

Here, we developed a minimal model of immune discrimination, by which we showed a possible mechanism of immune discrimination based on universal information about all antigen types, such as their concentration and input rapidness, and demonstrated temporal changes based on the history of antigen exposures. However, our current model considered antigen-induced responses of T cells that are specific to only one kind of antigens, and it did not include antigens that undergo self-renewal and can be eliminated by the immune system, such as pathogens. In previous studies, Domínguez-Hüttinger et al. and Christodoulides et al. have focused on the onset and therapy of atopic dermatitis and developed a mathematical model describing the interaction of pathogens, skin barrier integrity, and the innate/adaptive immune system.^65^^,^^66^ They revealed different phenotypes in patients derived from certain parameters (genetic risks) and suggested an effective treatment strategy based on the optimal control theory. To precisely describe immune discrimination for self-proliferating antigens, we should introduce antigen proliferation into our current model and consider its interaction with the immune system, which would potentially enable us to understand more complex immune responses, such as in the case of atopic dermatitis with immunological memory formation.

Finally, our model would also enable us to address how immune responses change throughout our life, as seen in the hygiene hypothesis. This hypothesis states that an unhygienic experience (experience of numerous infections) during early childhood prevents allergic diseases; on the contrary, hygienic environments raise their risk.^67^ The authenticity of this hypothesis is still controversial, but it suggests that antigen discrimination can be influenced by all previous exposures to multiple antigens, that is, personal hygiene. Our results on history-dependent discrimination, where immune responses to the same antigen input can be weakened by a certain antigen experience, is consistent with the hygiene hypothesis. Thus, our model might explain the difference in allergic risks based on individual antigen experiences. However, our current minimal model did not describe immune responses under multiple kinds of antigens based on the idea that responses to each antigen are dominantly determined by cells specific to each antigen, although immune cells specific to other antigens possibly contribute to the response under multiple types of antigens. Therefore, to validate the hygiene hypothesis, we need to expand our model into a form that is able to examine immune responses toward multiple antigens.

### Limitations of the study

The mathematical model developed in this study (the predictive immune memory model) is a minimal model that describes essential immune processes at the level of T cells. For simplicity, we only modeled the response to only one kind of antigens. In addition, antigen inputs are completely external input and antigens do not proliferate and they are not eliminated by immune responses in the current model. Thus, to examine more complex immune responses, such as atopic dermatitis and hygiene hypothesis, we need to expand our model into the model that describes responses to multiple kinds of antigens and the dynamics of antigens (their proliferation and elimination by immune responses).

## STAR★Methods

### Key resources table

**Table:** 

REAGENT or RESOURCE	SOURCE	IDENTIFIER
**Software and algorithms**		

MATLAB	https://jp.mathworks.com/products/matlab.html	RRID: SCR:001622 Version: 9.10.0.1602886 (R2021a)
Simulation codes	N/A	https://github.com/kyoshido1213/Simulations-of-Predictive-immune-memory-model

### Resource availability

#### Lead contact

Further information and requests should be directed to and will be fulfilled by the lead contact, Honda Naoki (nhonda@hiroshima-u.ac.jp).

#### Materials availability

The study did not generate any new materials.

### Experimental model and subject details

This study did not use experimental models and subjects.

### Method details

#### T cell population dynamics with different properties of T cell activation

The model was extended to include the effect of the dose-responses of T cell activation on the immune response as

$$\begin{array}{l} \frac{d}{dt}T_{conv}=-d_{c}T_{conv}+\frac{D_{c}}{1+s_{r}T_{reg}}T_{conv}+k_{c}\;T_{naive}\;q_{c}\left(a\right)+w_{c}\;M_{conv}\;q_{c}\left(a\right)-E_{c}T_{conv}\text{,} \end{array}$$

$$\begin{array}{l} \frac{d}{dt}T_{reg}=-d_{r}T_{reg}+\frac{D_{r}}{1+s_{c}T_{conv}}T_{reg}+k_{r}\;T_{naive}\;q_{r}\left(a\right)+w_{r}\;M_{reg}\;q_{r}\left(a\right)-E_{r}T_{reg}\text{,} \end{array}$$

where $q_{c}\left(a\right)$ and $q_{r}\left(a\right)$ represent the dose-responses of T_conv_ and T_reg_ cells, respectively, as described by the linear and Hill equation:

$$q_{i}\left(a\right)=\left\{ \begin{array}{l} \;a\;,\;when\;linear\;dose-response\\ Q_{i}\;\frac{a^{h_{i}}}{{K_{i}}^{h_{i}}+a^{h_{i}}},\;when\;sigmoidal\;and\;step-like\;dose-response\; \end{array}\right.$$

where $Q_{i}$, $K_{i}$ and $h_{i}$ (i∈{c,r}) indicate the amplitude, half-maximal effective antigen concentration, and Hill coefficient, respectively. Here, we considered three types of dose-response curves (linear, sigmoidal, and step-like). In the linear dose-response curve, the same equations were applied as Equations 1 and 2. In the sigmoidal and step-like dose-response curves, the Hill equation was applied, where $\left(h_{i},Q_{i},K_{i}\right)=\left(4,10,30\right)$ and (8,10,10), respectively. The same dose-response types were applied for both T_conv_ and T_reg_ cells in Figure 5 ($q_{c}\left(a\right)=q_{r}\left(a\right)$), whereas different types of dose-response were applied in Figures S5–S8 ($q_{c}\left(a\right)=q_{r}\left(a\right)$ or $q_{c}\left(a\right)\neq q_{r}\left(a\right)$).

#### T cell population dynamics with time delay in memory formation

The model was extended to include the effect of time delay in memory T cell formation.

$$\begin{array}{l} \frac{d}{dt}M_{conv}=-d_{mc}M_{conv}\left(t\right)+E_{c}T_{conv}\left(t-\tau_{delay}\right)\text{,} \end{array}$$

$$\begin{array}{l} \frac{d}{dt}M_{reg}=-d_{mr}M_{reg}\left(t\right)+E_{r}T_{reg}\left(t-\tau_{delay}\right)\text{,} \end{array}$$

where the prediction error is calculated based on the concentration of antigens and T cells at time $t-\tau_{delay}$:

$$\begin{array}{l} E_{c}=e_{c}{\left|a\left(t-\tau_{delay}\right)-m_{c}T_{conv}\left(t-\tau_{delay}\right)\right|}_{+}\text{,} \end{array}$$

$$\begin{array}{l} E_{r}=e_{r}{\left|f\left(T_{conv}\left(t-\tau_{delay}\right),a\left(t-\tau_{delay}\right)\right)-m_{r}T_{reg}\left(t-\tau_{delay}\right)\right|}_{+}\text{,} \end{array}$$

Biologically, it is a possibility that memory T cell generation based on the calculation of prediction error takes more time compared to T cell population dynamics stimulated by antigens.

### Quantification and statistical analysis

This study did not include statistical analysis and quantification.

### Additional resources

This study did not generate and contributed to a new website/forum, and it is not part of a clinical trial.

## Data Availability

No datasets were generated and analyzed during the current study. The predictive immune memory model was simulated using Matlab (R2021a) on GitHub (https://github.com/kyoshido1213/Simulations-of-Predictive-immune-memory-model). The parameters used in the simulation are provided in Table S1.
