# Supplementary material for: Adaptive discrimination between harmful and harmless antigens in the immune system by predictive coding
Source: iScience. 2022 Dec 7;26(1):105754. doi: 10.1016/j.isci.2022.105754 (PMC9804113; doi:10.1016/j.isci.2022.105754)
Supplement: Document S1. Figures S1–S8 and Table S1 [file mmc1.pdf]

iScience, Volume *26*

Supplemental information

Adaptive discrimination between harmful and harmless antigens in the immune system by predictive coding

Kana Yoshido and Honda Naoki

Supplemental information

Supplemental Tables:

Symbol	Name	Value	Unit
d_c	Death rate of T_{conv} cells	100	h^{-1}
d_r	Death rate of T_{reg} cells	10	h^{-1}
k_c	Rate of differentiation of T_{naive} cells to T_{conv} cells	1	$h^{-1}(mg/mL)^{-1}$
k_r	Rate of differentiation of T_{naive} cells to T_{reg} cells	0.01	$h^{-1}(mg/mL)^{-1}$
w_c	Rate of production of T_{conv} cells from memory T_{conv} cells	4	$h^{-1}(mg/mL)^{-1}$
w_r	Rate of production of T_{reg} cells from memory T_{reg} cells	0.4	$h^{-1}(mg/mL)^{-1}$
d_{mc}	Death rate of memory T_{conv} cells	0	h^{-1}
d_{mr}	Death rate of memory T_{reg} cells	0	h^{-1}
T_{naive}	Population of T_{naive} cells	1	$cells$
r_0	Basal rate of degradation rate of response	0.01	h^{-1}
r_s	Rate of suppression of response by T_{reg} cells	0.01	$h^{-1}(cells)^{-1}$
r_a	Rate of activation of response by T_{conv} cells	0.01	$h^{-1}(cells)^{-1}$
D_c	Proliferation rate of T_{conv} cells	1	h^{-1}
D_r	Proliferation rate of T_{reg} cells	1	h^{-1}
s_c	Rate of suppression of T_{reg} cell proliferation by T_{conv} cells	1	$cells^{-1}$
s_r	Rate of suppression of T_{conv} cell proliferation by T_{reg} cells	1	$cells^{-1}$
e_c	Basal rate of production of memory T_{conv} cells	0.0001	$h^{-1}(mg/mL)^{-1}$
e_r	Basal rate of production of memory T_{reg} cells	0.0003	$h^{-1}(mg/mL)^{-1}$
m_c	Coefficient of T_{conv} cells in prediction of antigen concentration	1	$(mg/mL)(cells)^{-1}$
m_r	Coefficient of T_{reg} cells in prediction of excessive immune response	1	$(mg/mL)(cells)^{-1}$
A_{max}	The amplitude of T_{conv} cell activation in prediction of excessive immune responses	100	mg/mL
K	Half-maximal effective T_{conv} cell population in prediction of excessive immune responses	1	$cells$

Table S1: List of parameter values for simulation, Related to STAR Methods.

Supplemental Figures:

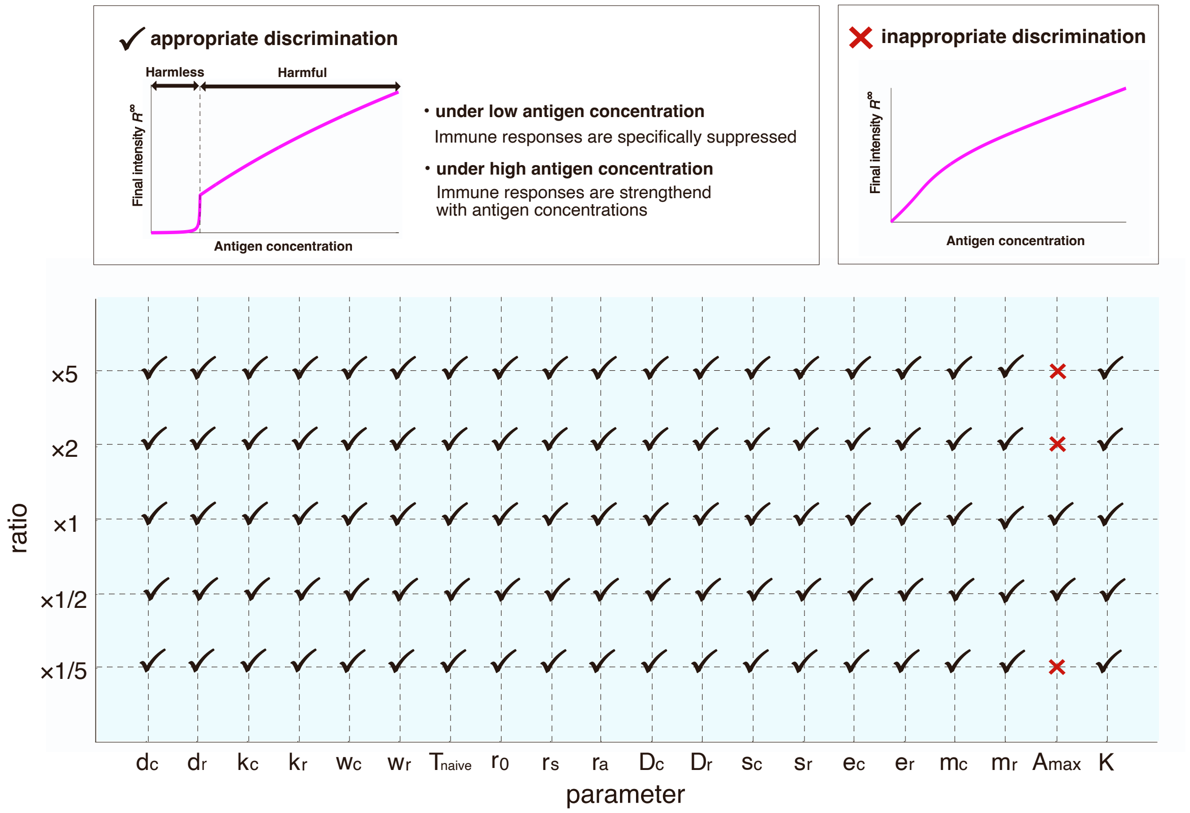

Figure S1. Parameter sensitivity analysis, Related to Figure 2.

Parameter sensitivity analysis of the predictive immune memory model was conducted by simulating concentration-dependent discrimination (as shown in Figure 2D), where one of the model parameters were multiplied by constants (1/5, 1/2, 1, 2, and 5). The check and cross marks indicate appropriate and inappropriate discrimination between harmful and harmless antigens, respectively. The upper diagrams indicate the definition of appropriate discrimination between harmful and harmless antigens. The representative results in each parameter condition are shown in Figure S2.

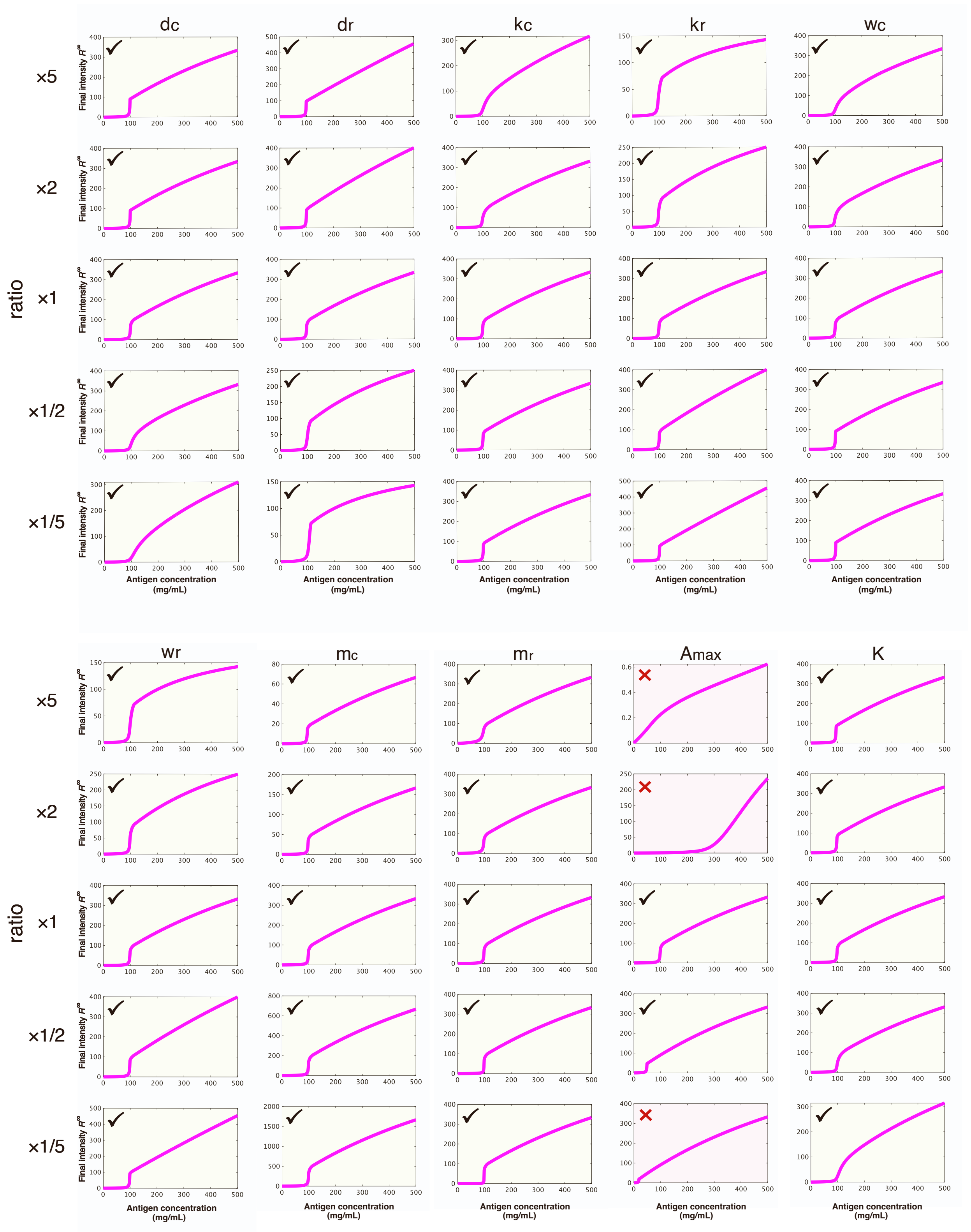

Figure S2. Parameter sensitivity analysis in each parameter condition, Related to Figure 2. Representative results of changes in the intensity of immune responses depending on antigen concentrations (as shown in Figure 2D) when varying one of the model parameters. Convergence value of the intensity R is plotted upon steady exposure to each antigen concentration. The check and cross marks indicate appropriate and

inappropriate discrimination between harmful and harmless antigens, respectively. The definition of appropriate discrimination between harmful and harmless antigens is shown in Figure S1.

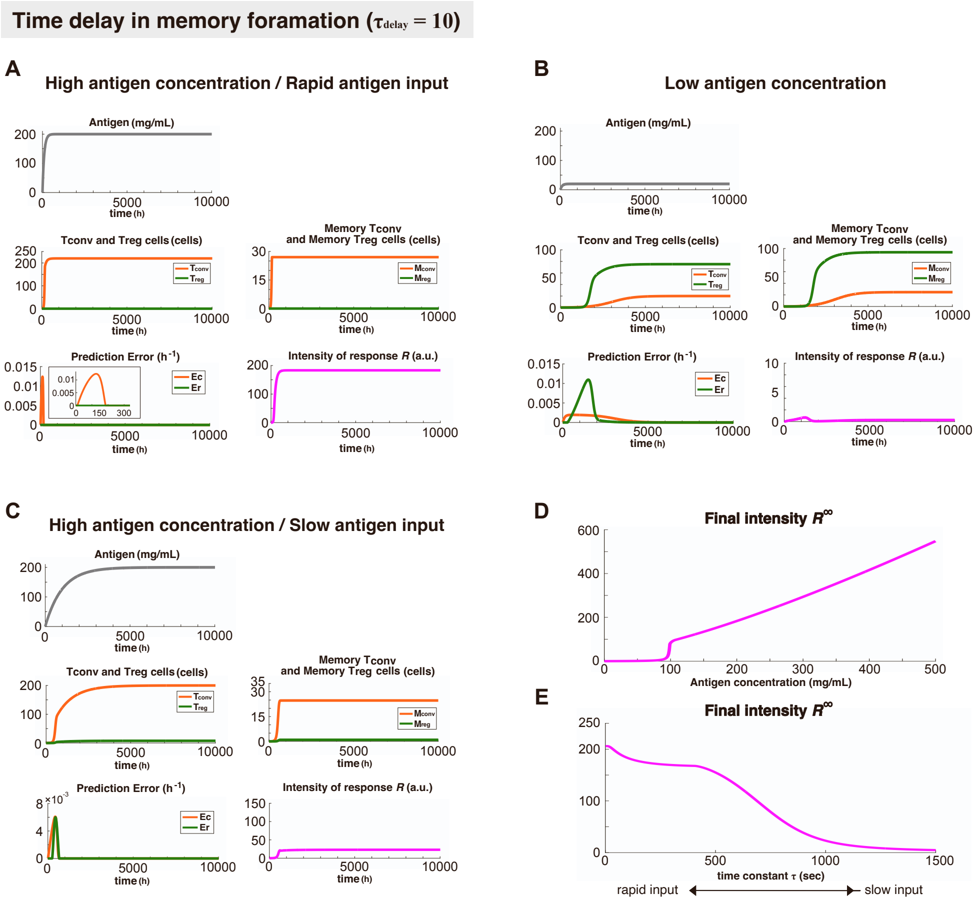

Figure S3. Antigen concentration-dependent and antigen input rapidness-dependent discrimination with time delay in memory formation (τ_{delay} = 10), Related to Figures 2 and 3.

Immune responses were simulated with time delay in memory formation $\tau_{delay} = 10$ (see the STAR Methods section). Antigens were administered as $a(t) = a_0(1 - e^{-t/\tau})$, where τ indicates the time constant of antigen input.

(A, B) Immune responses simulated with the exposure of (A) high and (B) low concentration of antigens. Insets show an enlarged view of the prediction error in the early phase.

(C) Immune responses simulated under slow inputs of high antigen concentration.

(D) Change in intensity of immune responses depending on antigen concentrations. Convergence value of the intensity R is plotted upon steady exposure to each antigen concentration.

(E) Change in intensity of immune responses depending on time constants of antigen administration. Convergence value of the intensity R is plotted at each time constant. High antigen concentrations ($a_0 = 200$) were administered.

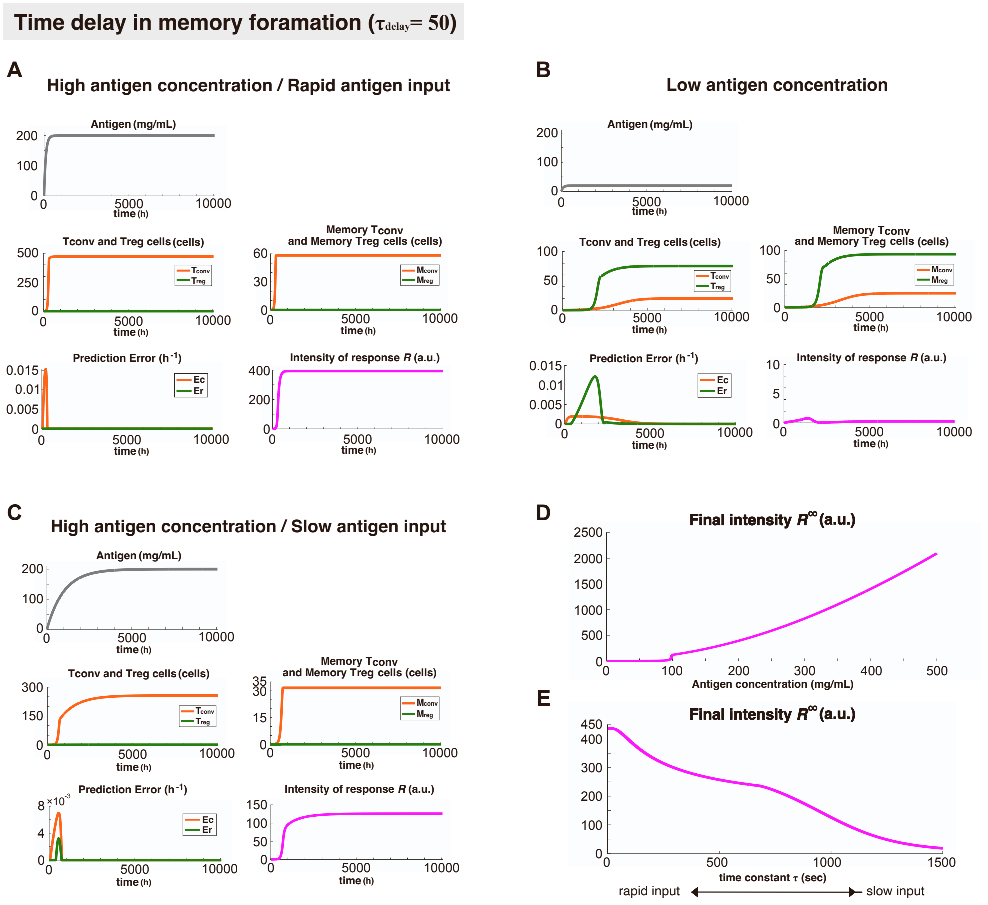

Figure S4. Antigen concentration-dependent and antigen input rapidness-dependent discrimination with time delay in memory formation ($\tau_{delay} = 50$), Related to Figures 2 and 3.

Immune responses were simulated with time delay in memory formation $\tau_{delay} = 50$ (see the STAR Methods section). Antigens were administered as $a(t) = a_0(1 - e^{-t/\tau})$, where τ indicates the time constant of antigen input.

(A, B) Immune responses simulated with the exposure of (A) high and (B) low concentration of antigens.

(C) Immune responses simulated under slow inputs of high antigen concentration.

(D) Change in intensity of immune responses depending on antigen concentrations. Convergence value of the intensity R is plotted upon steady exposure to each antigen concentration.

(E) Change in intensity of immune responses depending on time constants of antigen administration. Convergence value of the intensity R is plotted at each time constant. High antigen concentrations ($a_0 = 200$) were administered.

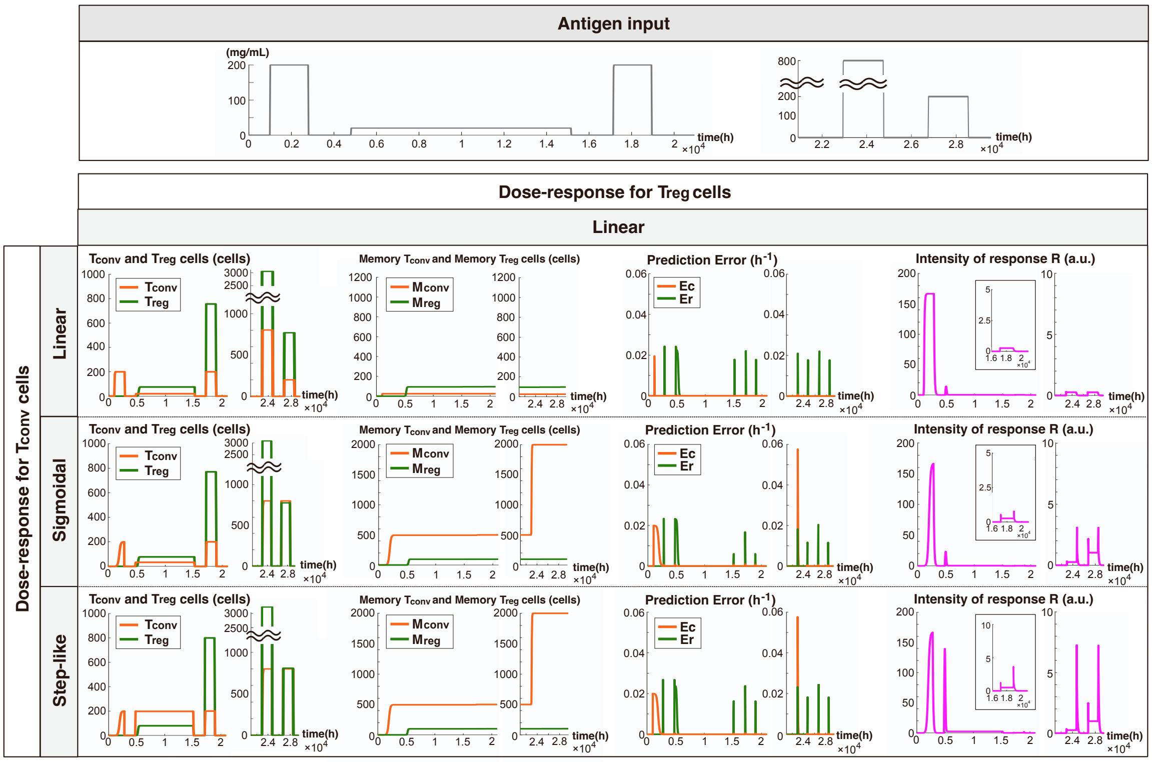

Figure S5. Simulations of therapeutic effect and its persistence when the dose-response of T_{reg} cells was linear, Related to Figure 5. Temporal changes in immune responses to a series of different antigen inputs (shown in each top panel) under all patterns of dose-response types for T_{conv} cells with linear T_{reg} cell activation. The first antigen input was high enough for allergy induction. The second input was applied for allergen immunotherapy. The third input was applied to check the therapeutic effect. The fourth antigen input was higher than the first one, and the fifth input was applied to examine the effect of the fourth antigen input. Insets show an enlarged view of the intensity of the responses during the third antigen input.

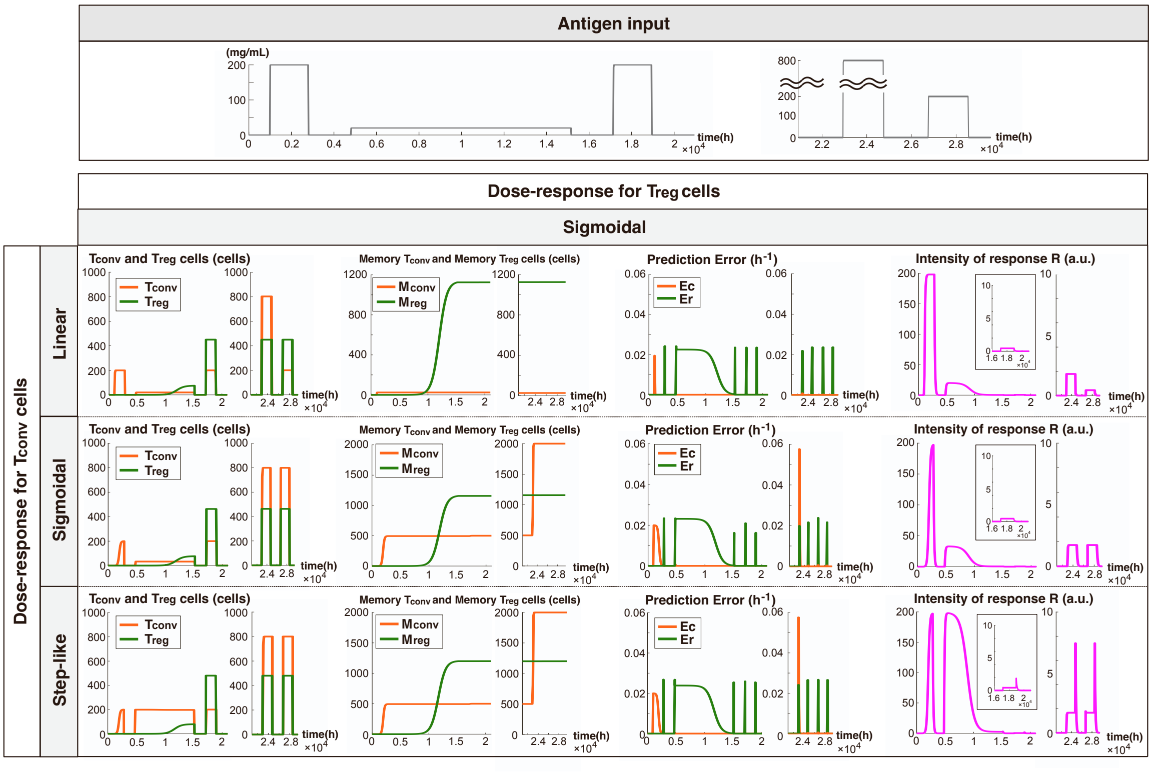

Figure S6. Simulations of therapeutic effect and its persistence when the dose-response of T_{reg} cells was sigmoidal, Related to Figure 5. Temporal changes in immune responses to a series of different antigen inputs (shown in each top panel) under all patterns of dose-response types for T_{conv} cells with sigmoidal T_{reg} cell activation. The first antigen input was high enough for allergy induction. The second input was applied for allergen immunotherapy. The third input was applied to check the therapeutic effect. The fourth antigen input was higher than the first one, and the fifth input was applied to examine the effect of the fourth antigen input. Insets show an enlarged view of the intensity of the responses during the third antigen input.

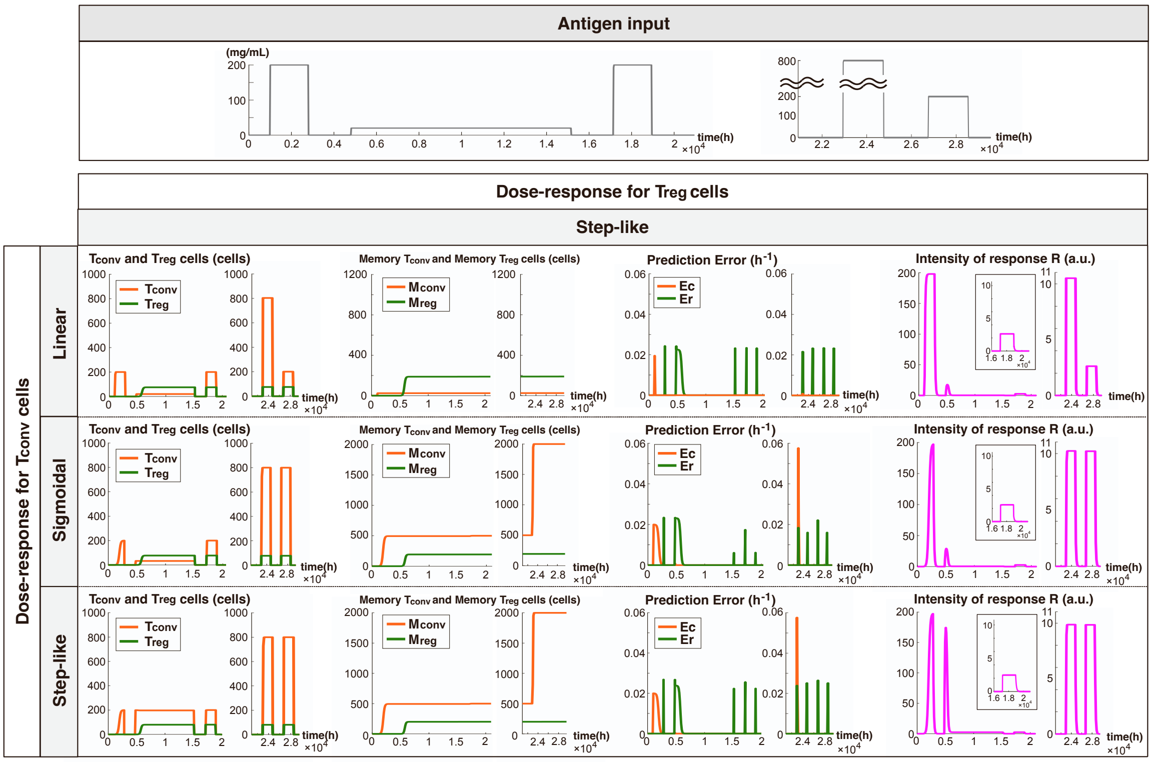

Figure S7. Simulations of therapeutic effect and its persistence when the dose-response of T_{reg} cells was step-like, Related to Figure 5. Temporal changes in immune responses to a series of different antigen inputs (shown in each top panel) under all patterns of dose-response types for T_{conv} cells with step-like T_{reg} cell activation. The first antigen input was high enough for allergy induction. The second input was applied for allergen immunotherapy. The third input was applied to check the therapeutic effect. The fourth antigen input was higher than the first one, and the fifth input was applied to examine the effect of the fourth antigen input. Insets show an enlarged view of the intensity of the responses during the third antigen input.

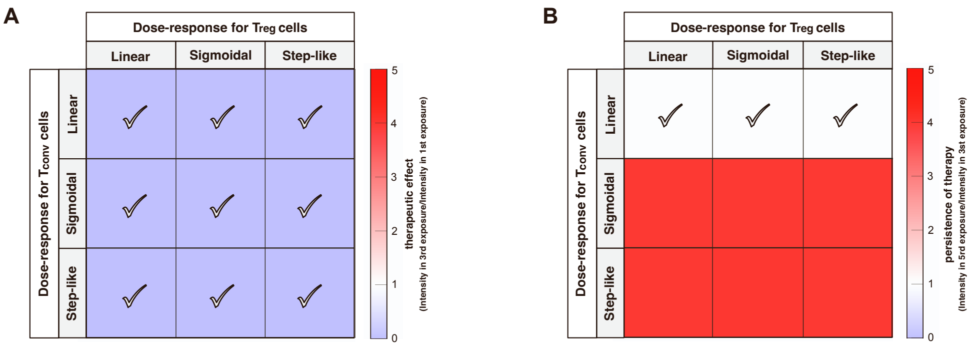

Figure S8. Therapeutic effect and its persistence in nine combinations of dose-responses of T cell activation, Related to Figure 5. (A) Heatmap showing the therapeutic effect in all combinations of dose-response types of T_{conv} and T_{reg} cell activation. The therapeutic effect was evaluated by the ratio of the maximum intensity of response to the third antigen input to that to the first antigen input. The check marks indicate the cases in which the therapeutic effect was less than 1; that is, allergen immunotherapy was successful.

(B) Heatmap showing the persistence of allergen immunotherapy in all combinations of dose-response types of T_{conv} and T_{reg} cell activation. Persistence was quantified by the ratio of the maximum intensity of response to the fifth antigen input to that to the third antigen input. Check marks indicate the cases where the quantified persistence was close to 1; that is, the effect of allergen immunotherapy persisted after the fourth antigen input. When calculating the maximum intensity on each antigen input, we ignored the transient peaks of intensity at the initiation and end of antigen input on the numerical simulation.
